# The Effect of Ethyl Esters of Linseed Oil on the Changes in the Fatty Acid Profile of Hair Coat Sebum, Blood Serum and Erythrocyte Membranes in Healthy Dogs

**DOI:** 10.3390/ani13142250

**Published:** 2023-07-09

**Authors:** Anna Wyrostek, Katarzyna Czyż, Ewa Sokoła-Wysoczańska, Bożena Patkowska-Sokoła, Wiesław Bielas

**Affiliations:** 1 Institute of Animal Breeding, Wrocław University of Environmental and Life Sciences, Chełmońskiego 38c, 51-630 Wrocław, Poland; katarzyna.czyz@upwr.edu.pl (K.C.); bozena.patkowska-sokola@upwr.edu.pl (B.P.-S.); 2 The Lumina Cordis Foundation, Szymanowskiego 2a, 51-609 Wrocław, Poland; 3 Department of Reproduction and Clinic of Farm Animal, Wroclaw University of Environmental and Life Sciences, 50-375 Wroclaw, Poland

**Keywords:** polyunsaturated fatty acids, PUFAs, omega-6, omega-3, dogs

## Abstract

**Simple Summary:**

Fatty acids from the omega-3 family play an important role in both human and animal organisms, but they are not synthesized in the body and must be provided with diet. This study examined an effect of ethyl esters of linseed oil rich in alpha-linolenic acid supplemented to healthy beagle dogs on the fatty acid profile of their blood serum, erythrocyte membranes and hair sebum. The treatment resulted in a decrease in the content of saturated fatty acids exhibiting adverse effects and an increase in the level of beneficial unsaturated acids, especially these from the omega-3 family.

**Abstract:**

The aim of this study was to determine the effect of supplementation with ethyl esters of linseed oil on the fatty acid profile in hair sebum, blood serum and erythrocyte membranes in healthy dogs. The material for the study included hair and blood samples of adult beagle dogs. The experiment was performed in two periods: summer and winter. Each time it lasted 16 weeks. During the first 8 weeks, the dogs received a supplement, the amount of which was determined individually so that the ratio of α-linolenic acid (ALA) to linoleic acid (LA) together in the feed and supplement was 1:1. Hair coat and blood samples were collected on days 0, 56 and 112; i.e., before the start of supplementation, after 8 weeks of supplementation and 8 weeks after supplementation was completed. The study included a determination of the fatty acid profile with a particular emphasis on polyunsaturated fatty acids (PUFAs) and the ratio of omega-6 to omega-3 in hair sebum, blood serum and erythrocyte membranes. As a result of supplementation, a significant decrease in saturated acids and an increase in unsaturated acids was observed in hair sebum both in summer and winter and especially in omega-3 fatty acids; i.e., α-linolenic (ALA) and its derivatives eicosapentaenoic acid (EPA) and docosahexaenoic acid (DHA). The same relationships were observed in blood serum and in erythrocyte cell membranes in all the studied periods. Additionally, 8 weeks after the end of supplementation, the level of polyunsaturated fatty acids was still higher compared to the period before supplementation. Moreover, the supplementation resulted in a favorable decrease in the ratio of omega-6 to omega-3 acids in the tested samples, which persisted even after the withdrawal period.

## 1. Introduction

Polyunsaturated fatty acids (PUFAs) of the omega-6 and omega-3 families, known as essential fatty acids (EFA), play a significant role in the proper development and functioning of the body [1,2,3,4]. Due to the lack of enzymes allowing for the formation of unsaturated bonds in the omega-3 and omega-6 positions; i.e., Δ-12 desaturase and Δ-15 desaturase, mammals are not able to synthesize the omega-6 and omega-3 series of PUFAs by themselves, and therefore they must be provided in the diet [4,5,6,7,8,9]. Both polyunsaturated fatty acids of the omega-6 and omega-3 families undergo enzymatic transformations in the organism consisting in the addition of double bonds (desaturation) under the influence of desaturases (Δ6, Δ5) and elongation of the hydrocarbon chain using elongases [7,10]. The products resulting from these enzymatic transformations; i.e., dihomo-γ-linolenic acid (DGLA), arachidonic acid (AA), adrenic acid from the omega-6 family, and eicosapentaenoic acid (EPA) and docosahexaenoic acid (DHA) from the omega-3 family, which are defined as long-chain polyunsaturated fatty acids (highly unsaturated fatty acids (HUFAs)), show biological activity. They are precursors of eicosanoids, which are tissue hormones showing a wide spectrum of biological activity [4,9]. The main difference is the fact that eicosanoids formed as a result of AA synthesis have a highly pro-inflammatory effect, and those formed by DGLA, EPA and DHA show a weak pro-inflammatory or anti-inflammatory effect [6,11,12,13].

Fatty acids from the exogenous omega-6 and omega-3 families together with the endogenous omega-9 family compete for the same enzymes in the conversion process [3,7,10,14,15]. However, regarding the most preferred substrates for Δ-6 desaturase, among them are alpha-linolenic acid, then linoleic acid and finally oleic acid [3,16,17,18].

An increase in alpha-linolenic acid (α-linolenic (ALA)) content in the diet increases the level of EPA and DHA in cell membranes and concurrently reduces AA content, but this process in dogs is considered inefficient [6,18,19,20]. Lenox [4] and Lenox and Bauer [21] reported that conversion in dogs, similarly as in humans, is less than 10%, and Twining et al. [9] even noted only about 5%. This process is considered more efficient in rats and other rodents [22]. The degree of ALA conversion to EPA and DHA can be also affected by the ratio of omega-6 to omega-3 acids; the ratio 4:1/3:1 is considered the most optimal for this process [7].

Numerous studies conducted on mammals have shown that a diet rich in linseed or linseed oil may cause an ALA, EPA, DPA (docosapentaenoic acid) and LA (linoleic acid) increase and an AA decrease in both plasma and erythrocyte membranes, but it does not affect the changes in DHA levels [18,19,23,24,25]. In turn, the use of fish oil, which is rich in EPA and DHA, causes their increase in erythrocyte membranes along with a simultaneous decrease in AA content [26,27].

Apart from compounds showing beneficial effects on the organism, linseed oil also contains anti-nutritive substances that may show negative effects [28,29,30]. At the same time, linseed oil is sensitive to light and atmospheric oxygen. Due to the presence of fat in the form of triglycerides, linseed oil stored improperly or for an excessive amount of time undergoes oxidation processes, which results in the loss of its pro-health properties. It is also characterized by a bitter taste, which is often considered unpleasant [31].

Ethyl esters of higher fatty acids obtained as a result of a transesterification reaction are characterized by better bioavailability to the organism in comparison to triglycerides. Ethyl esters of linseed oil are an excellent source of PUFAs, especially in about 50–60% of α-linolenic acid. Unlike linseed oil, they are free of harmful substances. Due to lower oxygen solubility in esters, they are also more durable and less susceptible to oxidation, epoxidation and peroxidation processes [31,32].

The aim of the study was to determine the effect of linseed oil ethyl esters on the changes in the fatty acid profile in the hair sebum, blood serum and erythrocyte membranes of healthy dogs.

## 2. Materials and Methods

### 2.1. Animals

The experiment was conducted on 8 healthy adult beagle dogs (3–5 years old, both sexes) weighing 9–13 kg and kept in the Department of Reproduction and Clinic of Farm Animals, Faculty of Veterinary Medicine, Wrocław University of Life Sciences. Throughout the whole period of the experiment, the dogs stayed in partially roofed outdoor enclosures with access to insulated shelters. The dogs had all the current vaccinations and were dewormed. The health status of the dogs was monitored daily, and the body weight was measured weekly. The food ration was set individually for each dog to ensure proper body weight and fitness. The dogs were fed twice a day (in the morning and in the afternoon). They had constant access to fresh water.

According to the opinion of the 2nd Local Ethical Committee for Experiments on Animals, Wroclaw University of Environmental and Life Sciences, Poland, the study did not require the approval of the Committee under the Act of 15 January 2015 on the protection of animals used for scientific and educational purposes [33].

### 2.2. Scheme of the Experiment

The experiment was conducted in two periods: summer (June to September 2015) and winter (November 2015 to February 2016). In both periods, the length of the experiment was 16 weeks, and the preparation period was an additional 8 weeks before the trial began. During the preparation period, the pet dry food that was used throughout the experiment was introduced. The average nutritional value of the food is shown in Table 1.

After the preparation period, all dogs received an additional supplement in the form of ethyl esters of linseed oil administered individually and orally using a syringe once a day after morning feeding for 8 weeks. In order to determine the correct amount of the supplement, the content of fatty acids in the food and supplement was determined by means of gas chromatography (Table 2). Synthesis of ethyl esters of linseed oil was conducted according to the technology developed at the University of Wrocław (Poland) [34]. The production technology and characteristics of ethyl ester used in this experiment are presented in the study by Sokoła-Wysoczańska et al. [32]. In brief, the technology involves transesterification of linseed oil (a mixture of triglycerides of omega-3, -6 and -9 fatty acids) with ethanol in the presence of a catalyst. The first stage of the process involves transesterification in an anaerobic atmosphere, then unreacted bioethanol is removed from the post-reaction mixture and the glycerin phase is separated from the raw ester phase in gravity separators. Then, the raw esters are subjected to cleaning via centrifugation followed by cleaning using a residual gas alcohol depot with nitrogen; the residual glycerin phase is subject to sedimentation. The glycerin phase is separated in the last step of the process.

The fatty acid profile was determined in the Laboratory of Chromatography and Meat Evaluation, Institute of Animal Breeding, Wrocław University of Environmental and Life Sciences, Poland. The amount of the supplement was determined so that the ratio of ALA to LA acids in total in the food and supplement was as close as possible to 1:1. The average amount of the supplement was 35 mL/day/dog. Hair coat and blood samples for the fatty acid profile, blood count and biochemical indices analysis were collected three times in both periods from all dogs participating in the experiment: week 0 (i.e., after 8 weeks of the preparation period), week 8 (i.e., after 8 weeks of the supplementation period) and week 16 (i.e., 8 weeks after the moment of discontinuing the supplement).

### 2.3. Biological Samples

The hair coat was collected using a surgical clipper (3M) on the left side at the level of the last rib by cutting square patches measuring 5 × 5 cm. The samples were packed in tightly closed containers and stored at −20 °C until the marking was carried out. Blood was collected from the cephalic vein of the front leg. Each time, 5 mL of blood was collected from one dog into the test tubes with potassium EDTA. After collection, the blood was centrifuged and divided into serum and morphotic elements and then was stored at −20 °C until the time of analysis.

### 2.4. Measurements and Analysis

Sebum fat was extracted with ether using the Soxhlet method. Fatty acid methyl esters were obtained using 2M KOH solution in methanol. The profile of the fatty acids in the obtained fat samples was determined using an Agilent 7890A gas chromatograph (Agilent Technologies, Santa Clara, CA, USA) with a flame ionization detector (FID) (Agilent Technologies, Santa Clara, CA, USA).

Fat present in serum and erythrocytes was extracted using the Folch method. Fatty acid methyl esters were obtained using 2M KOH solution in methanol. The fatty acid profile in the obtained fat samples was determined using an Agilent 7890A gas chromatograph (Agilent Technologies, Santa Clara, CA, USA) with an FID detector (Agilent Technologies, Santa Clara, CA, USA). The determinations were made in the following conditions: HP-88 capillary column (Agilent)—100 m long and 0.25 mm diameter at an initial temperature of 50 °C and with temperature increments of 3 °C/min to 220 °C; temperature of the dispenser—270 °C.

The identification of the obtained fatty acid peaks was performed via comparison with the retention times of the Sulpeco 37 fatty acid methyl ester standards from Sigma.

Blood count and biochemical indices were determined by the commercial veterinary laboratory VetLab sp. z o.o (Wroclaw, Poland).

### 2.5. Statistical Analysis

The obtained results were analyzed statistically by using the Statistica ver. 13.1 package. The normality of the data distribution was evaluated via the Shapiro–Wilk test. Results concerning the fatty acid profile in hair coat sebum, serum and erythrocyte membranes were evaluated by using a two-factor analysis of variance.

## 3. Results

The health status of dogs was constantly monitored during each experiment. Also, the body weight remained stable during the whole study. All dogs willingly consumed the food and supplement. They did not have any undesirable gastrointestinal clinical signs (e.g., diarrhea). They maintained proper physical condition throughout the whole period of the experiment.

The results of the blood count and biochemical indices are presented in Tables S1 and S2. A significant decrease in the ALT level in week 8 in summer and a significant difference in the haptoglobin content in week 0 between the summer and winter were noted. With the exception of fibrinogen, all parameters (both blood count and biochemistry) were within reference standards.

### 3.1. Sebum of the Hair Coat

The results for changes in fatty acid composition in the sebum are presented in Table 3. As a result of supplementation with ethyl esters of linseed oil, a significant decrease in the total sum of SFAs was observed in both experimental periods. In winter, a significant increase in the sum of MUFAs was observed. However, after 8 weeks of supplement withdrawal, the amounts of SFAs and MUFAs were significantly lower compared to the beginning of the experiment. A significant increase in total PUFAs at week 8 of supplementation was observed in both periods. At week 16, they were still at a significantly higher level compared to the beginning of the experiment.

Both in summer and winter, a significant increase in the contents of LA AA, ALA, EPA and DHA was observed at week 8 compared to the beginning of supplementation.

At week 16 of both periods, a significant increase in the contents of ALA and DHA was observed in relation to week 0. The contents of LA and AA in summer as well as AA and EPA in winter also increased.

The total sum of omega-6 and omega-3 series acids increased significantly as a result of supplementation (week 8) in both periods. In relation to the beginning of the experiment (week 0), their significantly higher level was observed at week 16 of the experiment, except for omega-6 in winter.

The ratio of omega-6 to omega-3 acids both in summer and in winter significantly decreased at week 8. At week 16, it was still at a significantly lower level compared to week 0.

Considering the differences in particular weeks between the seasons, significantly higher levels of PUFAs were noted in summer in weeks 0 and 8 as well as in the case of MUFAs in week 16. The contents of LA (week 8), AA (week 0), EPA and DHA were significantly higher in particular weeks in winter, while the opposite was true for ALA (weeks 0 and 16) and AA (weeks 8 and 16). The level of PUFAs in week 8 was significantly higher and in week 16 lower in winter compared to summer. The level of Omega-6 acids was significantly higher in winter in weeks 0 and 6, while the content of omega-3 acids in week 16 was higher in summer. Finally, the ratio of omega-6 to omega-3 acids was significantly higher in winter at the beginning of supplementation (Table 3).

### 3.2. Blood Serum

The results for changes in the fatty acid composition in the blood serum are presented in Table 4. A significant increase was observed for the total sum of MUFAs and PUFAs as well as a decrease in SFAs at week 8 of the experiment both in summer and winter. At week 16 of the summer experiment, a significant increase in MUFA content was observed compared to week 0.

At week 8 of the summer period of the experiment, a significant increase in the contents of LA, AA, ALA, EPA and DHA was observed. However, at week 16 of the same experiment, only the levels of EPA and DHA were still significantly higher compared to week 0.

At week 8 of the winter period of the experiment, the levels of ALA, EPA and DHA increased significantly, but at week 16 of the experiment only the level of DHA was significantly higher compared to week 0.

As a result of supplementation with ethyl esters of linseed oil (week 8), the level of omega-3 series acids increased significantly both in the experiments carried out in summer and winter, and at week 16 of the experiment, they were still at a significantly higher level in relation to week 0. The level of omega-6 series acids increased significantly only at week 8 of the summer period.

At week 8 of both experimental periods, the ratio of omega-6 to omega-3 acids decreased significantly. At week 16 of the experiment, its value increased compared to day 56, but it was still lower compared to week 0.

There were also significant differences between the seasons examined. The level of MUFAs at week 16 was significantly higher in summer. A similar trend was found for LA (week 8) as well as EPA. In turn, the levels of ALA (week 16), AA and DHA (weeks 0 and 8) were significantly higher in winter. The content of Omega-3 acids was significantly higher in winter at weeks 0 and 8, while the ratio of omega-6 to omega-3 was statistically higher in summer at week 0 (Table 4).

### 3.3. Erythrocyte Membranes

The results for changes in the fatty acid composition in the erythrocyte membranes are presented in Table 5. In both the summer and winter periods, a significant increase in PUFA content and a decrease in SFA at week 8 was observed. The PUFA level at week 16 did not return to the level at week 0, and in summer it was significantly higher. There were no significant differences in MUFA content between weeks 0 and 8, while at week 16 their level increased significantly in summer and in winter. No significant decrease in SFA content at week 16 was observed.

Both in the summer and winter periods of the experiment, a significant increase in the contents of AA, ALA, EPA and DHA was observed at week 8. At week 16 of both experimental periods, the levels of AA, ALA, EPA and DHA were significantly higher compared to week 0 of the experiment.

The sum of omega-6 acids increased significantly.

As a result of supplementation with ethyl esters (week 8), the sum of omega-3 series acids increased significantly in both periods of the experiment, and at week 16 it was still at a higher level compared to week 0.

In both experimental periods, the ratio of omega-6 and omega-3 acids at week 8 decreased significantly, and at week 16 it was still lower than at week 0.

Considering differences between summer and winter, in the case of erythrocyte membranes, only the level of MUFAs at week 16 was significantly higher in winter compared to summer (Table 5).

## 4. Discussion

Polyunsaturated fatty acids of the omega-6 and omega-3 series are essential for the proper development of the young organism and good health maintenance [1,2,3,4]. Due to their inability to be synthesized in an organism, linoleic acid (the precursor of the omega-6 series) and alpha-linolenic acid (the precursor of the omega-3 series) must be provided in the diet. Their main sources are plants and vegetable oils as well as fish oils for HUFAs (EPA and DHA) [3,10,14,35].

The authors of numerous studies have shown that 8-week supplementation with various oils (those of both vegetable and fish) brings significant changes in the fatty acid profile in erythrocyte membranes [26,27] or in blood serum [26,36,37,38], as in 10- [39] or 12-week supplementation [20,40,41,42,43]. The supplementation period in the experiments presented in this paper was also 8 weeks, and it was followed by statistically significant changes in the fatty acid profile of the sebum, serum and erythrocyte membranes. The increase in total PUFAs and the decrease in SFAs were particularly noticeable.

Stoeckel et al. [43] showed that there are no differences between the addition of a high-DHA and -EPA supplement to the food and the change in food to that high-DHA and -EPA one when it comes to changing the fatty acid profile. In addition, they showed that a decrease in omega-6 levels in erythrocyte membranes continued after discontinuation of the supplementation. The study also showed a decrease in the omega-6-to-omega-3 ratio from 30:1 to 12.5:1. A study by Hall et al. [44] also showed a decrease in the ratio in the group receiving fish oil from 18.4:1 to 2.2:1, which was similar to a study by LeBlanc et al. [42] in which the omega-6 ratio decreased from 17.6:1 to 4.4:1 in the group receiving fish oil and from 15.7:1 to 3.7:1 in the group receiving fish oil with vitamin E addition. The experiments also showed a statistically significant increase in the total sum of omega-3 fatty acids. The ratio of omega-6 to omega-3 in the sebum, blood serum and erythrocyte membranes, which persisted after withdrawal of the supplement, was also decreased.

Many authors have suggested that increasing ALA in the diet may cause an increase in EPA and DHA in the body membranes of dogs, but this process is considered inefficient [6,18,19,20]. Bauer [45] suggested that the conversion of ALA to EPA in dogs may be sufficient, while DHA is a more essential fatty acid in nutrition due the low conversion rate. The study showed that the addition of ethyl esters of linseed oil with a particularly high content of alpha-linolenic acid caused not only its increase but also an increase in eicosapentaenoic acid and docosahexaenoic acid levels; i.e., products resulting from its transformation.

The preference for ALA as a substrate by Δ6-desaturase may be associated with an increase in EPA and DHA and a decrease in the level of AA in the fatty acid profile of erythrocyte membranes. Additionally, the fact that LA is not converted makes it beneficial for the skin and hair coat [18]. In a study carried out by Jude et al. [26], the use of fish oil caused a significant increase in the omega-3 acid level in plasma and erythrocytes (especially DHA and EPA) and a decrease in AA. In humans, like in dogs, EPA is more readily incorporated into cell membranes [17]. A study conducted by LeBlanc et al. [42] showed a decrease in the level of AA and an increase in the levels of EPA and DHA in the control group receiving sunflower oil with high LA content and concurrently ALA in the amount of 1.2 g/kg of the diet. Bauer et al. [40] showed that ALA and EPA levels increased in the serum phospholipids of dogs after four days of supplementing linseeds. In addition, on day 84, the levels of DGLA and DPA n-3 increased, whereas the levels of AA and DPA n-6 decreased, but the level of DHA did not change. Dunbar, Bigley and Bauer [18] confirmed the previously obtained results with further studies, obtaining increases in ALA and EPA levels in plasma phospholipids after 84-day supplementation with linseed by 254 and 195%, respectively, without changes in DHA content. Additionally, the level of LA also increased. The authors confirmed that vegetable oils may be applied in dog nutrition in addition to fish oils. Also, in a study conducted by Purushothaman et al. [19], it was shown that the addition of linseed oil in the amount of 100 mL/kg of food used for a period of 3 weeks caused an increase in the levels of ALA, EPA and LA. However, no statistically significant changes in AA content were found. In addition, linseed oil has been shown to be a good source of ALA and EPA in dogs. Dahms et al. [38] used esters from algae oil with high DHA content in the experiment. After 8 weeks of supplementation with different levels of DHA, they noted a decrease in AA in each group above 500 mg/kg body weight/day and an increase in omega-3 from groups above 100 mg/kg body weight/day. Our study showed a significant increase in omega-3 series acids; i.e., ALA, EPA and DHA, in the hair sebum, blood serum and erythrocyte membranes of beagle dogs. An increase in omega-6 series acids; i.e., LA and AA, was reported in hair sebum along with an increase in AA in erythrocyte membranes.

A study conducted by Stoeckel et al. [43] showed that it took 18 weeks for EPA to return to its baseline state in the erythrocyte membranes of dogs and more than 18 weeks in the case of DHA. A study by Dahms et al. [38] also showed that 2 months after withdrawal, serum DHA levels were lower than after 9 months of supplementation but still higher than at the beginning of the experiment, suggesting that it was not completely rinsed out of the body. It was also shown in this study that ALA, EPA and DHA levels were still higher 8 weeks after withdrawal in the hair coat, serum and erythrocyte membranes of beagle dogs.

The fatty acid profile in erythrocyte membranes is considered to be a model for the membranes of other cells of an organism [20,46]. This may be related to the life expectancy of erythrocytes; in dogs, this period is about 17 weeks [43]. A study conducted by Stoeckel et al. [43] showed that the maximum level of erythrocyte membranes saturation with EPA and DHA acids was reached in the 8th week of supplementation, which was not confirmed in a study conducted by Jude et al. [26]. Kolanowski [46] also indicated the importance of the fatty acid profile in the blood serum, while according to Fuhrmann et al. [47], it is not reflected in the body. Bauer’s study [24] showed that the maximum EPA level in serum was already reached after 4 weeks of supplementation; in a study by Jude et al. [26], it was after 2–3 weeks. Both studies on erythrocyte membranes and serum studies confirmed the statement of Cao et al. [48], who suggested that in the case of short-term supplementation, blood plasma is a good reference for the level of fatty acids in human cells, whereas it is erythrocyte membranes in the case of long-term supplementation.

Studies on dogs indicate that they are also a model for human fatty acid metabolism studies due to their similarity in PUFA conversion [16,24,49].

The small number of dogs participating in the experiment and the lack of a control group were limitations of this study. However, the participation of dogs of one breed that were fed in the same way and kept in one environment makes the results more convincing. Further research including a larger number of healthy dogs from one breed and more experimental groups is needed in the future.

## 5. Conclusions

In conclusion, this study examined the effect of linseed oil ethyl esters on the changes in fatty acid profile in the hair sebum, blood serum and erythrocyte membranes in healthy dogs. As a result, a significant decrease in saturated fatty acids and an increase in unsaturated fatty acids in the fatty acid profiles of the hair coat sebum, blood serum and erythrocyte membranes in dogs participating in the experiment were observed as a result of ethyl ester of linseed oil supplementation. In particular, the contents of ALA and its derivatives; i.e., EPA and DHA, increased significantly, which contributed to the decrease in the ratio of omega-6 to omega-3 family acids. The conducted study may confirm that a diet in which the ratio of ALA to LA is 1:1 may facilitate the conversion of ALA to EPA and DHA, which is extremely important for the proper functioning of the body.

## Figures and Tables

**Table 1:** The average metabolic energy and nutrient content of food used in the experiment.

Metabolic Energy (kcal/100 g)	352.5
Dry matter (%)	90
Ash (%)	7
Protein (%)	25
Carbohydrates (%)	40.5
Fiber (%)	3
Fat (%)	14.5

**Table 2:** The average fatty acid content in the food used in the experiment and in the ethyl esters of linseed oil (%).

Fatty Acids	Content in the Food	Content in Ethyl Esters of Linseed Oil
Σ SFAs	29.95	7.87
Σ MUFAs	4.45	16.73
C18:2n6 linoleic acid (LA)	63.88	16.68
C18:3n3 α-linolenic acid (ALA)	1.72	58.72
Σ PUFAs	65.6	75.4
LA:ALA ratio	37.14:1	1:3.52

SFAs—saturated fatty acids; MUFAs—monounsaturated fatty acids; PUFAs—polyunsaturated fatty acids.

**Table 3:** Comparison of the fatty acid profile in the hair coat sebum of beagle dogs in weeks 0, 8 and 16 in summer and winter seasons (% of total FA pool).

Fatty Acids	Summer			Winter		
	Week 0	Week 8	Week 16	Week 0	Week 8	Week 16
Σ SFAs	34.768 ^a^_a_ ± 1.536	32.278 ^b^_a_ ± 1.866	33.042 ± 1.214	32.09 ^a^_b_ ± 1.545	29.646 ^b^_b_ ± 1.036	31.599 ± 1.911
Σ MUFAs	37.968 ± 1.778	39.499 ± 1.365	37.964 _a_ ± 1.563	36.096 ^A^ ± 1.658	39.175 ^B^ ± 1.497	35.383 ^A^_b_ ± 2.332
C18:2n6	9.135 ^Aa^ ± 0.922	11.354 ^B^_A_ ± 1.226	11.062 ^b^ ± 1.126	10.462 ^A^ ± 1.025	13.417 ^B^_B_ ± 1.010	12.085 ± 1.226
C18:3n3	4.347 ^A^_A_ ± 0.361	6.115 ^B^ ± 0.462	7.921 ^C^_A_ ± 0.917	3.157 ^A^_B_ ± 0.258	5.683 ^B^ ± 0.476	5.492 ^B^_B_ ± 0.515
C20:4n6	0.942 ^A^_A_ ± 0.062	3.171 ^B^_A_ ± 0.339	2.217 ^C^_A_ ± 0.169	1.314 ^Aa^_B_ ± 0.110	2.675 ^B^_B_ ± 0.193	1.596 ^Ab^_B_ ± 0.091
C20:5n3	0.692 ^A^ ± 0.059	1.356 ^B^_A_ ± 0.094	0.769 ^A^_A_ ± 0.073	0.815 ^A^ ± 0.078	1.658 ^B^_B_ ± 0.115	1.124 ^C^_B_ ± 0.123
C22:6n3	0.713 ^A^_A_ ± 0.067	1.485 ^B^_A_ ± 0.145	1.251 ^C^_A_ ± 0.108	1.014 ^A^_B_ ± 0.094	1.725 ^B^_B_ ± 0.138	1.564 ^B^_B_ ± 0.115
Σ PUFAs	24.234 ^A^ ± 1.259	26.738 ^B^_A_ ± 1.379	27.958 ^B^_A_ ± 1.429	22.908 ^A^ ± 1.239	29.868 ^B^_B_ ± 0.901	25.599 ^C^_B_ ± 1.225
n-6	13.911 ^A^_a_ ± 0.967	16.264 ^B^_A_ ± 1.347	16.395 ^B^ ± 1.067	15.591 ^A^_b_ ± 1.232	19.199 ^B^_B_ ± 0.812	15.226 ^A^ ± 1.192
n-3	5.752 ^A^ ± 0.308	8.956 ^B^ ± 0.458	9.941 ^C^_A_ ± 0.857	4.986 ^A^ ± 0.304	9.066 ^Ba^ ± 0.439	8.18 ^Bb^_B_ ± 0.606
n-6/n-3	2.425 ^A^_A_ ± 0.215	1.822 ^B^ ± 0.194	1.659 ^B^ ± 0.177	3.141 ^A^_B_ ± 0.366	2.122 ^B^ ± 0.126	1.874 ^B^ ± 0.232

Different superscripts indicate statistical differences between the weeks; different subscripts indicate statistical differences between the seasons (a, b—*p* < 0.05; A, B, C—*p* < 0.01). SFAs—saturated fatty acids; MUFAs—monounsaturated fatty acids; PUFAs—polyunsaturated fatty acids; C18:2n6—linoleic acid; C18:3n3—α-linolenic acid; C20:4n6—arachidonic acid; C20:5n3—eicosapentaenoic acid; C22:6n3—docosahexaenoic acid; n-6—omega-6; n-3—omega-3.

**Table 4:** Comparison of the serum fatty acid profile of beagle dogs in weeks 0, 8 and 16 in summer and winter periods (% of total FAs pool).

Fatty Acids	Summer			Winter		
	Week 0	Week 8	Week 16	Week 0	Week 8	Week 16
Σ SFAs	44.908 ^A^ ± 1.551	37.816 ^B^ ± 2.163	41.393 ± 2.297	44.835 ^A^ ± 3.198	38.451 ^B^ ± 3.153	43.287 ^A^ ± 2.595
Σ MUFAs	12.462 ^A^ ± 0.908	15.807 ^B^ ± 1.149	16.09 ^B^_a_ ± 1.105	13.034 ^A^ ± 0.778	16.65 ^B^ ± 1.373	14.373 ^A^_b_ ± 0.8530
C18:2n6	18.771 ^a^ ± 1.157	21.815 ^b^_A_ ± 1.571	19.259 ± 1.960	16.514 ± 2.020	18.447 _B_ ± 1.994	16.819 ± 1.412
C18:3n3	0.391 ^A^ ± 0.040	1.026 ^B^ ± 0.088	0.433 ^A^_a_ ± 0.035	0.485 ^A^ ± 0.048	1.115 ^B^ ± 0.108	0.532 ^A^_b_ ± 0.029
C20:4n6	8.627 ^A^_A_ ± 0.899	11.174 ^B^_a_ ± 1.109	8.958 ^A^_A_ ± 0.840	12.752 _B_ ± 1.388	13.123 _b_ ± 1.438	12.619 _B_ ± 1.169
C20:5n3	1.036 ^A^_A_ ± 0.093	2.394 ^B^_A_ ± 0.176	1.277 ^C^_A_ ± 0.092	0.725 ^A^_B_ ± 0.081	1.518 ^B^_B_ ± 0.104	0.824 ^A^_B_ ± 0.041
C22:6n3	0.825 ^A^_A_ ± 0.067	2.341 ^B^_A_ ± 0.211	1.663 ^C^ ± 0.181	1.521 ^A^_B_ ± 0.191	3.672 ^B^_B_ ± 0.307	1.947 ^C^ ± 0.193
Σ PUFAs	34.087 ^A^ ± 1.394	43.769 ^B^ ± 1.510	36.200 ^A^ ± 2.305	37.015 ^A^ ± 2.782	43.531 ^B^ ± 3.193	38.033 ^A^ ± 1.383
n-6	30.349 ^A^ ± 1.386	36.375 ^Ba^ ± 1.610	31.325 ^b^ ± 2.263	32.223 ± 2.716	34.825 ± 3.252	32.477 ± 1.177
n-3	3.225 ^A^_A_ ± 0.192	6.798 ^B^_A_ ± 0.279	4.324 ^C^ ± 0.182	3.801 ^A^_B_ ± 0.171	7.450 ^B^_B_ ± 0.346	4.434 ^C^ ± 0.259
n-6/n-3	9.442 ^A^_a_ ± 0.739	5.362 ^B^ ± 0.387	7.252 ^C^ ± 0.557	8.492 ^A^_b_ ± 0.783	4.685 ^B^ ± 0.494	7.335 ^C^ ± 0.271

Different superscripts indicate statistical differences between the weeks; different subscripts indicate statistical differences between the seasons (a, b—*p* < 0.05; A, B, C—*p* < 0.01). SFAs—saturated fatty acids; MUFAs—monounsaturated fatty acids; PUFAs—polyunsaturated fatty acids; C18:2n6—linoleic acid; C18:3n3—α-linolenic acid; C20:4n6—arachidonic acid; C20:5n3—eicosapentaenoic acid; C22:6n3—docosahexaenoic acid; n-6—omega-6; n-3—omega-3.

**Table 5:** Comparison of fatty acid profile in erythrocyte membranes of beagle dogs in weeks 0, 8 and 16 in summer and winter periods.

Fatty Acids	Summer			Winter		
	Week 0	Week 8	Week 16	Week 0	Week 8	Week 16
Σ SFAs	47.09 ^A^ ± 2.785	38.485 ^B^ ± 2.845	40.972 ^B^ ± 1.944	45.123 ^A^ ± 1.742	39.076 ^B^ ± 2.055	39.946 ^B^ ± 1.954
Σ MUFAs	11.297 ^A^ ± 0.943	12.329 ^a^ ± 0.776	13.871 ^Bb^_A_ ± 0.405	12.314 ^A^ ± 0.726	12.133 ^A^ ± 1.155	15.962 ^B^_B_ ± 1.333
C18:2n6	23.885 ± 2.903	24.817 ± 2.007	25.264 ± 1.823	23.271 ± 1.991	24.734 ± 1.674	22.578 ± 2.416
C18:3n3	1.133 ^A^ ± 0.125	1.657 ^B^ ± 0.139	1.379 ^C^ ± 0.115	1.056 ^A^ ± 0.078	1.511 ^B^ ± 0.116	1.438 ^B^ ± 0.115
C20:4n6	5.568 ^A^ ± 0.462	7.652 ^Ba^ ± 0.621	6.735 ^Bb^ ± 0.159	5.258 ^Aa^ ± 0.460	7.123 ^Ba^ ± 0.673	6.240 ^b^ ± 0.640
C20:5n3	0.723 ^A^ ± 0.070	1.741 ^B^ ± 0.169	1.438 ^C^ ± 0.329	0.812 ^A^ ± 0.063	1.825 ^B^ ± 0.171	1.519 ^C^ ± 0.140
C22:6n3	0.941 ^A^ ± 0.077	1.522 ^B^ ± 0.108	1.289 ^C^ ± 0.104	1.012 ^A^ ± 0.089	1.579 ^B^ ± 0.136	1.313 ^C^ ± 0.102
Σ PUFAs	39.078 ^Aa^ ± 2.979	44.952 ^B^ ± 1.854	43.045 ^b^ ± 1.761	38.343 ^A^ ± 2.296	44.188 ^Ba^ ± 1.719	40.422 ^b^ ± 2.765
n-6	34.02 ^a^ ± 2.980	37.427 ^b^ ± 1.865	36.811 ± 1.678	33.541 ^a^ ± 2.192	37.294 ^b^ ± 1.846	34.140 ± 2.787
n-3	4.083 ^A^ ± 0.187	6.242 ^B^ ± 0.267	5.412 ^C^ ± 0.192	4.209 ^A^ ± 0.199	6.308 ^B^ ± 0.323	5.668 ^C^ ± 0.260
n-6/n-3	8.355 ^A^ ± 0.911	6.005 ^B^ ± 0.375	6.805 ^B^ ± 0.297	7.971 ^A^ ± 0.401	5.931 ^B^ ± 0.490	6.036 ^B^ ± 0.587

Different superscripts indicate statistical differences between the weeks; different subscripts indicate statistical differences between the seasons (a, b—*p* < 0.05; A, B, C—*p* < 0.01). SFAs—saturated fatty acids; MUFAs—monounsaturated fatty acids; PUFAs—polyunsaturated fatty acids; C18:2n6—linoleic acid; C18:3n3—α-linolenic acid; C20:4n6—arachidonic acid; C20:5n3—eicosapentaenoic acid; C22:6n3—docosahexaenoic acid; n-6—omega-6; n-3—omega-3.

## Data Availability

No new data was created.

## Supplementary Materials

The following supporting information can be downloaded at: https://www.mdpi.com/article/10.3390/ani13142250/s1.
