# Supplementary material for: The Effect of Ethyl Esters of Linseed Oil on the Changes in the Fatty Acid Profile of Hair Coat Sebum, Blood Serum and Erythrocyte Membranes in Healthy Dogs
Source: Animals (Basel). 2023 Jul 9;13(14):2250. doi: 10.3390/ani13142250 (PMC10375989; doi:10.3390/ani13142250)
Supplement: Supplementary file 1 [file animals-13-02250-s001.zip › animals-2421084-supplementary.pdf]

Table S1. Blood count results of beagle dogs in week 0, 8 and 16, in summer and winter season (mean values ± SD)

Specification	Summer			Winter			Reference values*
	**Week 0.**	**Week 8.**	**Week 16.**	**Week 0.**	**Week 8.**	**Week 16.**	
WBC	11.99 ±3.0	13.68 ±2.6	11.34 ±2.7	10.34 ±2.1	11.39 ±2.8	10.23 ±3.2	6.0-16.9
Limf#	2.15 ±0.1	2.60 ±0.5	2.31 ±0.7	1.89 ±0.4	2.13 ±0.9	2.34 ±1.1	0.8-5.1
Mon#	0.63 ±0.1	0.70 ±0.1	0.58 ±0.1	0.56 ±0.1	0.66 ±0.3	0.79 ±0.5	0-1.8
Gran#	9.21 ±3.0	10.35 ±1.9	8.49 ±2.2	7.89 ±1.8	8.35 ±2.4	7.35 ±2.4	4.0-12.6
%Limf	18.73 ±4.0	18.40 ±2.3	17.83 ±2.3	18.30 ±2.6	18.96 ±4.4	21.35 ±3.4	12.0-30.0
%Mon	5.50 ±1.5	5.00 ±1.1	5.54 ±1.7	5.55 ±1.5	6.00 ±1.7	6.81 ±1.8	2.0-9.0
%Gran	75.74 ±5.2	76.35 ±3.1	76.11 ±4.3	75.90 ±3.8	75.04 ±5.8	70.71 ±6.4	60.0-83.0
RBC	7.10 ±0.6	6.89 ±0.47	7.32 ±0.56	7.21 ±0.68	7.16 ±0.88	6.88 ±0.49	5.50-8.50
HGB	164.75 ±14.8	155.88 ±11.4	163.13 ±13.1	162.00 ±15.4	156.38 ±23.1	155.50 ±12.9	110-190
HCT	48.26 ±4.8	46.63 ±2.9	48.90 ±3.9	48.58 ±4.2	47.84 ±3.3	45.79 ±3.6	39.0-56.0
MCV	68.00 ±3.1	67.85 ±2.7	67.69 ±2.0	68.06 ±2.4	68.05 ±3.3	66.66 ±2.4	62.0-72.0
MCH	23.16 ±0.9	22.63 ±0.9	22.79 ±0.6	22.91 ±0.7	22.43 ±1.5	22.55 ±0.8	20.0-25.0
MCHC	341.13 ±5.9	333.63 ±5.4	337.25 ±5.7	337.00 ±6.3	341.88 ±7.6	339.00 ±5.5	300-380
RDW	13.30 ±0.6	14.48 ±0.8	13.90 ±0.4	14.00 ±0.8	13.41 ±1.4	13.63 ±1.0	11.0-15.5
PLT	371.00 ±93.4	384.88 ±70.2	340.00 ±74.4	376.13 ±71.9	302.75 ±78.2	290.75 ±85.0	117-460
MPV	8.03 ±1.0	8.14 ±1.2	8.08 ±0.8	7.83 ±0.4	7.85 ±0.8	8.60 ±1.1	7.0-12.9

* reference values according to VetLab sp. z o.o.

Table S2. Blood biochemistry results of beagle dogs in week 0, 8 and 16, in summer and winter season (mean values ± SD)

Specification	Summer			Winter			Reference values*
	Week 0.	Week 8.	Week 16.	Week 0.	Week 8.	Week 16.	
AST(GOT) [U/l]	36.3 ±8.9	32.5 ±7.7	30.5 ±5.9	31.1 ±7.2	25.4 ±7.0	30.1 ±5.3	1.00-76.0
ALT(GPT) [U/l]	53.6[a] ±16.2	36.7[b] ±5.9	39.2 ±10.4	50.9 ±10.0	34.1 ±6.1	39.2 ±7.2	1.00-80.0
Urea [mmol/l]	5.68 ±1.24	5.21 ±1.41	5.61 ±1.44	5.16 ±1.11	4.1 ±0.65	4.91 ±1.23	3.30-8.30
Fibrinogen [g/l]	4.4 ±1.31	3.25 ±0.80	4.15 ±1.08	4.04 ±0.49	3.74 ±0.89	3.54 ±0.49	1.20-2.90
Creatinine [µmol/ml]	81.3 ±9.29	80.21 ±8.17	78.85 ±11.04	70.83 ±8.11	69.49 ±7.60	69.2 ±11.05	35-132
Haptoglobin [mg/ml]	1.16_a ±0.71	1.37 ±1.09	1.26 ±0.45	2.38_b ±0.87	1.85 ±0.95	2.01 ±0.41	no data
Triglycerides [mmol/l]	0.98 ±0.29	0.77 ±±0.39	0.82 ±0.36	0.67 ±0.26	0.59 ±0.20	0.63 ±0.24	0.11-1.71
Cholesterol [mmol/l]	6.11 ±1.08	5.26 0.94	5.81 ±1.22	5.22 ±0.78	4.75 ±0.93	5.16 ±0.73	3.10-8.60
LDL [mmol/l]	2.43 ±1.44	2.17 ±0.57	2.34 ±0.94	2.93 ±1.25	2.15 ±1.36	2.33 ±2.1	no data
HDL [mmol/l]	3.97 ±0.45	4.88 ±1.51	4.38 ±0.88	4.6 ±1.60	5.05 ±1.77	4.64 ±1.21	no data

* reference values according to VetLab sp. z o.o.

Different superscripts indicate statistical differences between the weeks: a, b $p< 0.05$

Different subscripts indicate statistical differences between the seasons: a, b $p< 0.05$
